# IL‐3 is essential for ICOS‐L stabilization on mast cells, and sustains the IL‐33‐induced RORγt^+^ T_reg_ generation via enhanced IL‐6 induction

**DOI:** 10.1111/imm.13305

**Published:** 2021-01-27

**Authors:** Sebastian Drube, Sylvia Müller, Franziska Weber, Philine Wegner, Romy Böttcher‐Loschinski, Matthias Gaestel, Andreas Hutloff, Thomas Kamradt, Nico Andreas

**Affiliations:** ^1^ Institut für Immunologie Universitätsklinikum Jena Jena Germany; ^2^ Medizinische Klinik 5 Universitätsklinikum Erlangen Erlangen Germany; ^3^ Institut für Zellbiochemie Medizinische Hochschule Hannover Hannover Germany; ^4^ Institut für Immunologie und Institut für Klinische Molekularbiologie Universitätsklinikum Schleswig‐Holstein Kiel Germany

**Keywords:** IL‐3, IL‐33, immune homeostasis, mast cells, T_regs_

## Abstract

IL‐33 is a member of the IL‐1 family. By binding to its receptor ST2 (IL‐33R) on mast cells, IL‐33 induces the MyD88‐dependent activation of the TAK1‐IKK2 signalling module resulting in activation of the MAP kinases p38, JNK1/2 and ERK1/2, and of NFκB. Depending on the kinases activated in these pathways, the IL‐33‐induced signalling is essential for production of IL‐6 or IL‐2. This was shown to control the dichotomy between RORγt^+^ and Helios^+^ T_regs_, respectively. SCF, the ligand of c‐Kit (CD117), can enhance these effects. Here, we show that IL‐3, another growth factor for mast cells, is essential for the expression of ICOS‐L on BMMCs, and costimulation with IL‐3 potentiated the IL‐33‐induced IL‐6 production similar to SCF. In contrast to the enhanced IL‐2 production by SCF‐induced modulation of the IL‐33 signalling, IL‐3 blocked the production of IL‐2. Consequently, IL‐3 shifted the IL‐33‐induced T_reg_ dichotomy towards RORγt^+^ T_regs_ at the expense of RORγt^−^ Helios^+^ T_regs_. However, ICOS‐L expression was downregulated by IL‐33. In line with that, ICOS‐L did not play any important role in the T_reg_ modulation by IL‐3/IL‐33‐activated mast cells. These findings demonstrate that different from the mast cell growth factor SCF, IL‐3 can alter the IL‐33‐induced and mast cell‐dependent regulation of T_reg_ subpopulations by modulating mast cell‐derived cytokine profiles.

## Graphical abstract

IL‐3 is essential for the expression of ICOS‐L on BMMCs, which was downregulated by co‐stimulation with IL‐33. While IL‐3 potentiated the IL‐33‐induced IL‐6 production by mast cells, it blocked the IL‐33‐induced production of IL‐2 and thereby, shifted the IL‐33‐induced Treg dichotomy towards RORγt^+^ T_regs_ at the expense of RORγt^−^ Helios^+^ T_regs_. Our findings demonstrate that IL‐3 can alter the IL‐33‐induced and mast cell‐dependent regulation of Treg subpopulations by modulating mast cell‐derived cytokine profiles.

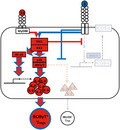

**Abbreviations**
BMMC: bone marrow‐derived mast cells
DUSP5: dual‐specificity phosphatase 5
ERK: extracellular signal‐regulated kinases
FcεRI: Fcε receptor I
FoxP3: Forkhead box protein P3
G6PI: Glucose‐6‐phosphate isomerase
ICOS: Inducible T‐cell costimulator
ICOS‐L: ICOS ligand
IKK: IκB kinase
IL‐2R: Interleukin‐2 receptor
IL‐33R: Interleukin‐33 receptor
IκB: inhibitor of NF‐κB
JNK: c‐Jun N‐terminal kinases
MAPK: Mitogen‐activated protein kinase
MAPKAPK: MAPK‐activated protein kinases
MK2/3: MAPKAPK2/MAPKAPK3
MKKs: MAPK kinase
NFκB: nuclear factor kappa B
RAR: retinoic acid receptor
RORγt: RAR‐related orphan receptor gamma, isoform t
SCF: stem cell factor
TAK1: Transforming growth factor beta‐activated kinase 1
TPL2: Tumour progression locus 2
Tregs: regulatory T cells

## INTRODUCTION

Mast cells are critical modulators of the innate and adaptive immune responses by recruitment of leucocytes^1^, ^2^, ^3^ and by support of the maturation of DCs and T lymphocytes.^4^, ^5^ Mast cells can induce tissue inflammation^6^ and therefore, contribute to immune responses against pathogens^4^ or to the development of allergic diseases^7^ and autoimmunity.^8^, ^9^, ^10^ Therefore, understanding of mast cell activities is fundamental to influence the balance of tissue‐protecting and tissue‐destructing processes. Hallmarks of mast cells are the strong expression of the high‐affinity Fcε‐receptor I (FcεRI), c‐Kit (CD117) and the IL‐3 receptor (CD123). FcεRI represents the best‐characterized mechanism of mast cell activation, which results in the production and the release of inflammatory mediators by degranulation. Besides CD117 activation by the stem cell factor (SCF), IL‐3 activation of CD123 signalling is essential for mast cell differentiation and survival *in vivo* and *in vitro*.^11^ Activation of IL‐3R induces the activation of the IKK2 signalling,^12^ and MAP kinases such as ERK1/2, JNK and p38.^12^ All of these signals are involved in mast cell differentiation, survival and proliferation.^12^, ^13^ Besides FcεRI, the Toll‐like/IL‐1 receptor (TIR) family member IL‐33R is also highly expressed on mast cells.^14^ IL‐33, the ligand for the IL‐33R, is an IL‐1 family member, which induces the activation of the MyD88‐TAK1‐IKK2 signalling module. This results in activation of the p38‐MAPKAPK2/ MAPKAPK3 (p38‐MK2/3) signalling module, ERK1/2, JNK1/2 and NFκB.^1^, ^15^, ^16^ Both the IL‐33‐induced activation of NFκB and of the p38‐MK2/3 signalling module result in the transcriptional and translational regulation of the cytokine production.^1^ IL‐33 is released upon damage of endothelial and epithelial cells,^17^ and thus, IL‐33 is termed an alarmin, which signals the loss of integrity of cellular layers with barrier functions. Mast cells represent a major cellular sensor for such damage‐released IL‐33 in peripheral tissues and are a component of the first line of response to cell injury.^18^ Although such mechanisms result in allergic inflammation,^2^, ^18^ IL‐33‐activated mast cells also downregulate inflammatory responses in peripheral tissues by inducing the formation of regulatory T cells (T_regs_).^19^ T_regs_ are dominant immune‐suppressive T cells characterized by the transcription factor FoxP3. One stable subpopulation of FoxP3^+^ T_regs_ is characterized by the expression of the transcription factor RORγt^20^ and can predominantly be found in gut‐associated lymphoid tissues (GALT) or on mucosal surface.^20^ Recently, we found that IL‐33‐activated mast cells control the dichotomy between RORγt^+^ and HELIOS^+^ T_regs_ via the production of IL‐6.^21^ Growth factors such as IL‐3 and SCF modulate IL‐33‐induced cytokine responses in mast cells.^1^, ^12^, ^22^, ^23^

ICOS is a coactivating receptor expressed on Treg cells and is essential for the maintenance of FoxP3.^24^ On the other hand, the ligand of ICOS, ICOS‐L, can be induced on mast cells.^25^ Stimulation of Tregs by ICOS‐L promotes Treg induction and stability.^26^ General blockade of the ICOS‐L/ICOS costimulatory axis is of immunosuppressive character *in vivo* as blocking ICOS‐L reduces the severity of symptoms in G6PI‐induced arthritis^27^ or collagen‐induced arthritis^28^ resulting in inhibited Th effector responses.^29^ However, the lack of ICOS signalling specifically in Tregs worsens the symptoms of diabetes and releases the suppression of IBD.^24^, ^30^

In the presented work, we aimed to investigate the influence of IL‐3 on the IL‐33‐induced generation of RORγt^+^ T_regs_. We show that IL‐3 is a main factor to stabilize the expression of ICOS‐L on mast cells. Besides that, IL‐3 potentiated the IL‐33‐induced IL‐6 response. In summary, IL‐3 exhibits potential immunoregulatory capacities via ICOS‐L stabilization, but more importantly dramatically enhanced the IL‐33‐induced production of IL‐6, and thus promotes the shift of T_reg_ subset characteristic towards RORγt^+^ T_regs_.

## MATERIALS AND METHODS

### Mice

We used sex‐ and age‐matched *wt* and *mk2^−/−^/mk3^−/−^* double‐deficient (*mk2/3^ko^*) C57BL/6.^31^ Mice were maintained according to the guidelines of the institutional and governmental committees for animal welfare of the Animal Research Facility of the Jena University Hospital and Medizinische Hochschule Hannover, Institut für Zellbiochemie. We isolated organs from sacrificed *wt* C57BL/6 and *mk2^−/−^/3^−/−^* double‐deficient (*mk2/3^ko^*) mice. Organ isolations are approved by the Thüringer Landesamt für Lebensmittelsicherheit und Verbraucherschutz; Bad Langensalza. All mice were used under the license twz‐36‐2017 for the Institute of Immunology, Jena.

### BMMC generation

BMMCs were generated by the standard protocol.^1^ Briefly, isolated bone marrow was cultured in IMDM (PAA) supplemented with 10% FCS, 100 U/ml penicillin, 100 mg/ml streptomycin, 50 mM 2‐mercaptoethanol and supernatant of a X63Ag‐653 BPV‐rmIL‐3 stably transfected cell line equivalent to 20 ng/ml IL‐3. After 4 weeks, cultures were tested by flow cytometry. BMMC cultures were considered as mature when cultures consisted of 95% c‐Kit^+^/ FcεRI^+^ BMMCs.

### Cell sorting

Isolated spleens were homogenized through a 100‐µm mesh. Afterwards, erythrocytes were lysed with a buffer containing 4.15 g NH_4_Cl, 0.51 g KHCO_3_, 14.6 mg EDTA, pH 7.2–7.4. Cells were washed with PBA‐E (PBS, 5 mg/ml BSA, 10 mM NaN_3_, 2 mM EDTA). Regulatory T cells were isolated by staining the splenocytes with FITC‐anti‐CD25 (Miltenyi) and APC‐Cy7‐anti‐CD4 (Thermo Fisher). To exclude dead cells, splenocytes were treated with propidium iodide (PI) prior to sorting. PI^−^CD4^+^CD25^+^ splenocytes (T_regs_) were sorted with an upgraded ARIA I sorter (ARIA III upgrade) by using the Diva8 software (BD Bioscience).

### Treatment with inhibitors

BMMCs (10^6^ cells/ ml) were seeded in IL‐3‐free media and were subsequently treated with inhibitors SP600125 (1 µM (0.01% DMSO), Merck), SB203580 (1 µM (0.01% DMSO), Merck), UO126 (5 µM (0.1% DMSO), Merck), IKK‐16/IKKiVII (1 µM (0.1% DMSO), Merck), PF3644022 (10 µM (0.1% DMSO), Merck) or its representative DMSO concentration as a control. Upon 30 min of incubation, all further stimulations were added to the culture as indicated.

### Coculture

For cocultures, 100 000 BMMCs were seeded with 25 000 MHC‐II^−^CD4^+^CD25^+^T_reg_ in the presence of 1.5 µg/ml soluble anti‐mouse CD3ε (clone 2C11, Institute for Immunology) in the presence of IL‐33 (50 ng/ml, Peprotech), with or without IL‐3 (50 ng/ml, Peprotech), with or without SCF (50 ng/ml, Peprotech) in a 96‐well plate for 3 days. In some experiments (as indicated in the figures), we added recombinant IL‐2 (50 ng/ml, Peprotech), a blocking anti‐ICOS‐L antibody (10 µg/ml, clone MIL‐5733^27^), a blocking anti‐IL‐3 antibody (10 µg/ml, clone MP2‐8F8, Biolegend), or a blocking IL‐6 antibody (10 µg/ml, clone MP5‐20F3, Biolegend) to the cocultures. Coculture experiments were performed with biological replicates (indicated in the figures).

### Flow cytometry

BMMCs were harvested and washed with PBA‐E (PBS, 5 mg/ml, BSA, 10 mM NaN3, 2 mM EDTA). To block unspecific binding prior to antibody staining, BMMCs were preincubated in the presence of rat IgG (Jackson) at 4°C for 5 min. For the determination of the purity of the BMMC cultures, cells were stained with PE‐anti‐CD117 (Biolegend) and FITC‐anti‐FcεRI (Biolegend). Furthermore, we used FITC‐anti‐CD117 (Biolegend), APC‐Cy7‐anti‐CD117 (Biolegend) and PE‐anti‐CD275/ICOS‐L (Biolegend) as indicated in the figures and figure legends. All antibodies were diluted in PBA‐E and were incubated at 4°C. After 20 min, BMMCs were washed with PBA‐E and analysed by using LSR II flow cytometer (BD). For the analysis of T_reg_/BMMC cocultures, we used the FoxP3 Transcription Factor Staining Buffer Set (Thermo Fisher). According to the manufacturer's protocol, cells were washed and fixed and were intracellularly stained with APC‐Cy7‐anti‐CD4 (Thermo Fisher), PacBlue‐anti‐FoxP3 (Thermo Fisher), PE‐anti‐RORγt (Thermo Fisher) and PerCP‐eFluor 710‐anti‐HELIOS (Thermo Fisher). Subsequently, cells were analysed with the LSR II flow cytometer (BD) and data were analysed by using FlowJo 10 (Treestar Inc., Ashland, OR).

### Stimulation, lysis and immunoblotting

BMMCs (10^6^ cells/ml) were seeded in IL‐3‐free media and were stimulated with IL‐3 (Peprotech, 50 ng/ml). BMMC lysis was performed in a buffer containing 20 mM HEPES, pH 7.5; 10 mM EGTA; 40 mM β‐glycerophosphate; 2.5 mM MgCl_2_; 2 mM orthovanadate; 1 mM dithiothreitol; 20 µg/ml aprotinin; and 20 µg/ml leupeptin supplemented with 1% Triton. Afterwards, protein concentration was determined with the BCA kit (Pierce). Prior to separation on 10% sodium dodecyl sulphate (SDS)–Laemmli gels, samples were boiled in 6× Laemmli buffer. Subsequently, proteins were transferred onto nitrocellulose membranes (Biostep) by electroblotting. Blocked membranes (with dry milk) were incubated overnight with anti‐pT202/Y204‐ERK1/2; anti‐pT183/Y185‐JNK1/2; anti‐pT180/Y182‐p38; or the respective anti‐total antibodies (all from Cell Signalling), were washed in 0.1% Tween/TBS and finally incubated with secondary anti‐rabbit Ig or anti‐mouse Ig (Thermo Fisher Scientific) coupled with HRP‐conjugated secondary antibodies. The detection of the phosphorylated/ non‐phosphorylated proteins was performed with ECL reagent (Pierce). The intensities of the Western blots were quantified with the ImageJ software (Fiji; Freeware). We performed the Western blots with three biological replicates. The phosphorylation blots were normalized to the total protein Western blots, whereas the control (unstimulated samples) was set as 1.

### ELISA

BMMCs were seeded (10^6^ cells/ml) in IL‐3‐free medium. After 1 h, BMMCs were preincubated with DMSO (vehicle) or inhibitors (all from Merck) for 30 min. The inhibitors were used as indicated in the figures and the figure legends. Afterwards, cells were prestimulated with IL‐3 (50 ng/ml) for 30 min and then stimulated with IL‐33 (50 ng/ml) (both from Peprotech) for 24 h. Supernatants were collected and analysed for IL‐6 or IL‐2 by using matched paired antibodies (eBioscience). For ELISA experiments, we used biological replicates separated into at least three technical replicates.

### RT‐PCR

Upon mast cell stimulation, total RNA was extracted with the TRIzol reagent (Invitrogen) according to the manufacturer's instruction. 500 ng RNA was reversely transcribed using Oligo(dt)18 primer and SuperScript™ IV Reverse Transcriptase (Thermo Fisher Scientific) according to the manufacturer's instruction. RT‐qPCR was performed using 2 μl of template cDNA, primers for *Icosl* (Mouse ICOS ligand qPCR primer pair, Sino Biological, MP200220) or glyceraldehyde‐3‐phosphate‐dehydrogenase (*Gapdh*, forward 5′ TTGGCCGTATTGGGCGCCTG 3′, reverse 5′ CACCCTTCAAGTGGGCCCCG 3′) as housekeeping gene. Reaction volume was calculated to 20 μl, and PowerUp SYBR Green Master Mix (Applied Biosystems) was used for detection. Samples were analysed in duplicates in the AB 7500 Real‐Time PCR System (Applied Biosystems) according to the manufacturer's instructions. Expression levels of *Icosl* transcripts were normalized to *Gapdh*.

### Statistical analysis

Statistical analysis was performed with IBM SPSS Statistics version 20.0 (IBM, Ehningen, Germany). Results are shown as the mean of measurements ± SEM. Significance was assessed by Student's *t‐*test (*p < 0.05; **p < 0.01; ***p < 0.001).

## RESULTS

### IL‐3 induced an ERK1/2‐dependent maintenance of the surface expression of ICOS‐L on mast cells

**Figure 1:**
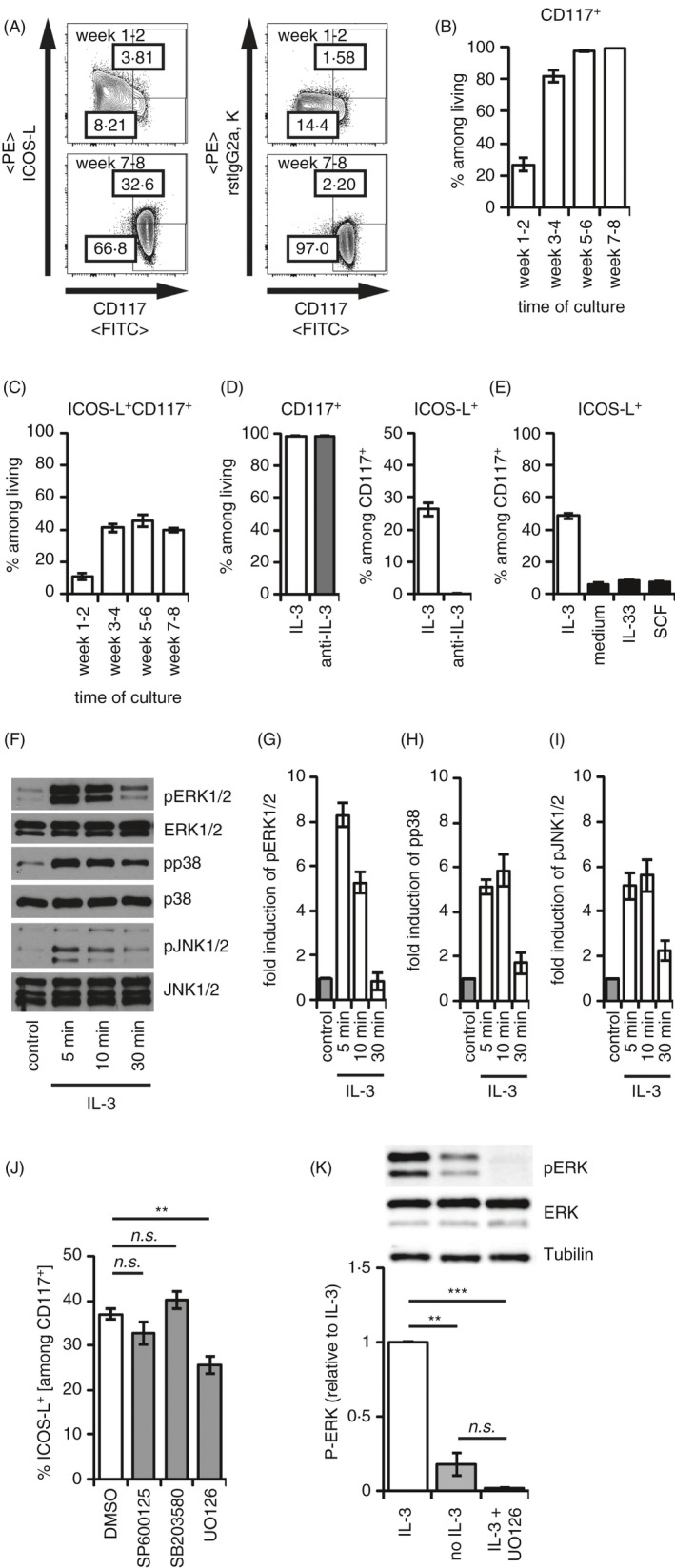
IL‐3 stabilizes ICOS‐L on mast cells via ERK signalling. Bone marrow‐derived mast cells (BMMCs) were cultured as indicated, and ICOS‐L expression was assessed on CD117^+^ BMMCs by flow cytometry. (A‐C) BMMCs were generated from total bone marrow cells of C57BL/6 mice in medium containing supernatant of a X63Ag‐653 BPV‐rmIL‐3 stably transfected cell line equivalent to 20 ng/ml IL‐3. Differentiation was monitored over 8 weeks (*n* = 12 independent biological replicates). FACS plots in (A) show representative results after 1–2 weeks or after 7–8 weeks of BMMC generation. Generation of CD117^+^ BMMCs is summarized in (B). ICOS‐L expression among CD117^+^ BMMCs is summarized in (C). (D) BMMCs were cultured in the presence of supernatant of a X63Ag‐653 BPV‐rmIL‐3 stably transfected cell line equivalent to 20 ng/ml IL‐3 with or without a blocking anti‐IL‐3 antibody (10 µg/ml) for 24 h. CD117^+^ BMMCs and ICOS‐L^+^ cells among CD117+ BMMCs are summarized in the diagrams (*n* = 6 independent biological replicates). (E) BMMCs were cultured in medium alone or medium containing either 50 ng/ml recombinant IL‐3, 50 ng/ml recombinant IL‐33 or 50 ng/ml recombinant SCF for 24 h. Frequencies of ICOS‐L^+^ cells among CD117^+^ BMMCs are summarized (*n* = 3 independent biological replicates). (F‐I) BMMCs were starved from IL‐3 for 60‐min prior stimulation with 50 ng/ml IL‐3 for the indicated time periods. Phosphorylation of MAP kinases ERK1/2, JNK1/2 and p38 was analysed by Western blotting. Representative Western blot bands are shown in (F). Phosphorylation of MAP kinases ERK1/2 (G), p38 (H) and JNK1/2 (I) was quantified (*n* = 4 biologically independent experiments). (J) BMMCs were cultured in the presence of recombinant IL‐3 and DMSO or with JNK inhibitor (SP600125, *n* = 3 biological replicates), p38 inhibitor (SB203580, *n* = 6 biological replicates), or ERK inhibitor (UO126, *n* = 6 biological replicates) for 24 h. Subsequently, frequencies of ICOS‐L^+^ cells among CD117^+^ BMMCs were analysed and summarized in the diagram. (K) BMMCs were starved from IL‐3 for 30 min and incubated in the presence of DMSO or UO126 for further 30 min. Subsequently, cells were cultured in medium alone or in the presence of 50 ng/ml IL‐3 for 24 h. Phosphorylation of MAP kinases ERK1/2 was analysed by Western blotting. Representative Western blot bands are shown, and quantification of ERK phosphorylation is summarized in the diagram below (*n* = 3 independent biological replicates). Diagrams show average values of all experiments ± SEM. Statistics were done with Student's *t‐*test: *^n.s^*. not significant; * *p* < 0.05; **p < 0.01, ***p < 0.001

Similar to dendritic cells, mast cells have been reported to express ICOS‐L.^32^ To characterize the regulation of ICOS‐L on mast cells *in vitro*, we monitored the expression of ICOS‐L during BMMC generation. A representative gating strategy is shown in Figure S1A. ICOS‐L is upregulated after the 3^rd^ and 4^th^ week of BMMC differentiation and remains stably expressed on CD117^+^ BMMCs (Figure 1A‐C). To exclude a secondary effect of the IL‐3‐conditioned supernatant used for mast cell generation, we blocked IL‐3 in these cultures by the addition of a monoclonal anti‐IL‐3 antibody. The downregulation of ICOS‐L upon blockade of IL‐3 indicated that on fully differentiated mast cells, the ICOS‐L expression is maintained particularly by IL‐3 (Figure 1D). Upon removal of the IL‐3‐conditioned supernatant from the mast cell culture by washing the cells, ICOS‐L expression was downregulated on mast cells upon reculture in medium without IL‐3 supplementation after 24 h (Figure 1E). In the absence of IL‐3, IL‐33 or SCF was insufficient to maintain the expression of ICOS‐L on mast cells (Figure 1E). Next, we determined the signalling pathways, which maintain ICOS‐L on mast cells. IL‐3 stimulation of mast cells induced activation of MAP kinases ERK, p38 and JNK (Figure 1F‐I). To investigate the role of these kinases in the IL‐3‐mediated stabilization of ICOS‐L on the surface, we inhibited the kinase activities with the help of well‐described inhibitors, namely SP600125 (JNK inhibitor^33^), SB203580 (p38 inhibitor^34^) and UO126 (ERK inhibitor^35^). However, only ERK inhibition resulted in a significant decrease in ICOS‐L surface expression (Figure 1J). Depletion of IL‐3 signals did similarly result in reduced ERK activation, indicating that ERK is a major signalling node important for the IL‐3‐induced ICOS‐L expression on the surface of mast cells (Figure 1K).

### IL‐3 promotes the IL‐33‐induced and mast cell‐dependent formation of RORγt^+^ T_regs_ independently from ICOS‐L

**Figure 2:**
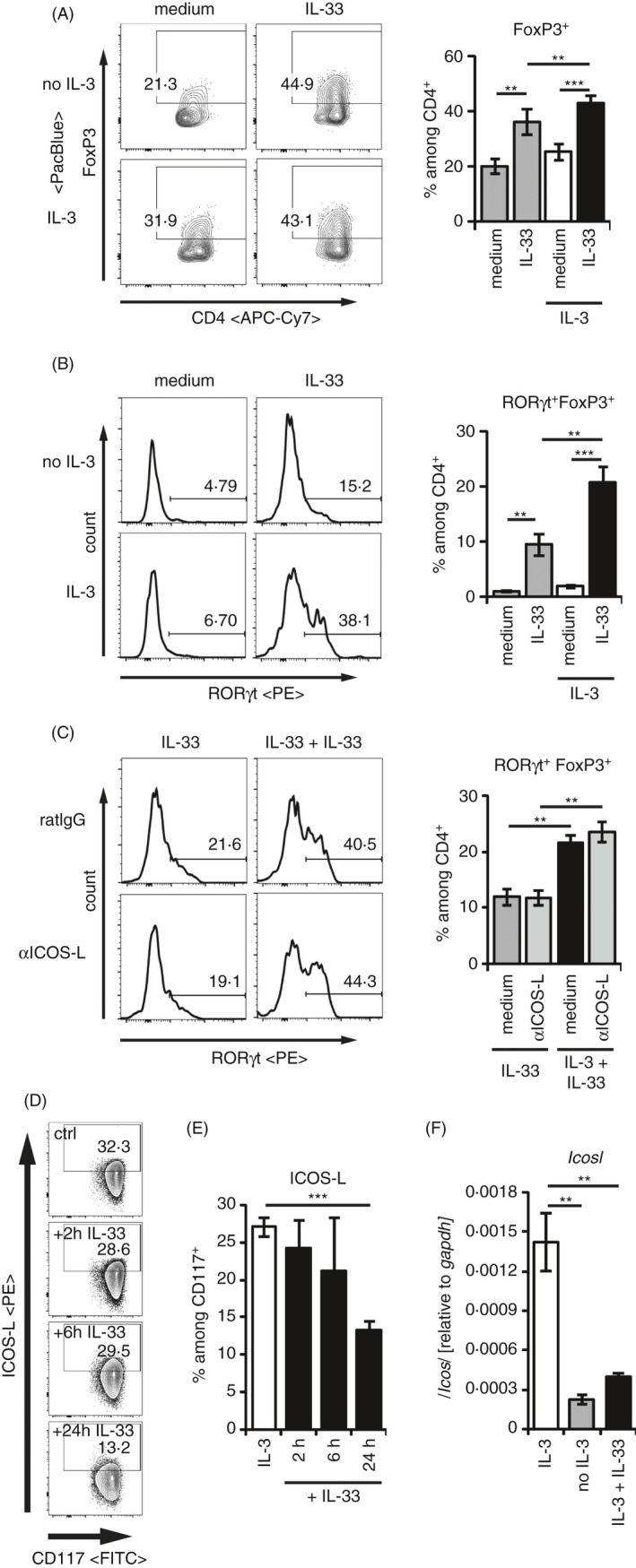
IL‐3 enhances the IL‐33‐induced RORγt^+^ T_reg_ development independent from ICOS‐L. (A‐C) CD25^+^CD4^+^ splenic T_regs_ were cocultured with BMMCs in medium ± 50 ng/ml recombinant IL‐33 alone, or in the presence of 50 ng/ml recombinant IL‐3 ± 50 ng/ml recombinant IL‐33 for 3 days. Transcription factor expression of FoxP3 among CD4^+^, and RORγt among FoxP3^+^CD4^+^T_regs_ was analysed by flow cytometry. Representative FACS plots and histograms are shown in the right panels. Diagrams on the right summarize data from separate experiments representing BMMCs from *n* > 10 biologically independent donors. (A) FoxP3^+^ cells among CD4+ Th cells. (B) RORγt^+^FoxP3^+^ expression among CD4^+^Th cells. (C) RORγt^+^FoxP3^+^ expression among CD4^+^Th cells in the presence of blocking anti‐ICOS‐L antibody. (D, E) BMMCs were cultured in the presence of recombinant 50 ng/ml IL‐3 (*n* = 12 different donors) and 50 ng/ml IL‐33 for 2 (*n* = 2 different donors), 6 (*n* = 2 different donors) or 24 h (*n* = 12 different donors). Frequencies of ICOS‐L^+^ cells were analysed among CD117^+^ BMMCs by flow cytometry. (D) Representative FACS plots. (E) Data from all cultures of BMMCs are summarized. (F) BMMCs were cultured in medium (*n* = 6), medium containing 50 ng/ml recombinant IL‐3 alone (*n* = 6) or with additional 50 ng/ml recombinant IL‐33 (*n* = 3). Transcript levels of *Icosl* and *Gapdh* were quantified by qRT‐PCR after 24 h. Relative expressions of *Icosl* related to *Gapdh* are summarized from the indicated numbers of independent biological replicates. Diagrams show average values of all experiments ± SEM. Statistics were done with Student's *t‐*test: *^n.s^*. not significant; * *p* < 0.05; **p < 0.01, ***p < 0.001

We recently showed that IL‐33‐stimulated BMMCs induce RORγt^+^ T_regs_ from RORγt^−^ T_regs_.^21^ We analysed Tregs in the cocultures with mast cells as represented by the gating strategy shown in Figure S1B. When we added IL‐3 to these cultures, we did not observe any effect on the overall T_reg_ frequencies among CD4^+^Th cells (Figure 2A). However, we observed a strong increase in the capacities of BMMCs to induce RORγt^+^ T_regs_ in the presence of IL‐3 (Figure 2B). While the presence of IL‐3 did slightly improve the survival of the mast cells in these cocultures (Figure S2A,B), the overall T_reg_ frequencies remained comparable in the presence and absence of IL‐3 (Figure 2A). Therefore, we conclude that the increase in RORγt^+^ T_regs_ was not mediated by an improved mast cell survival.

Recently, ICOS‐L on dendritic cells has been linked to the formation of RORγt^+^ T_regs_.^36^ On the other hand, T_regs_ have been shown to express ICOS, but not ICOS‐L.^37^ Because IL‐3 stabilizes ICOS‐L and addition of IL‐3 to the IL‐33 stimulated cocultures induced more RORγt^+^ T_regs_, we stimulated T_regs_ and BMMCs with IL‐33 or with IL‐3 and IL‐33 in the presence of a blocking ICOS‐L antibody. Unexpectedly, anti‐ICOS‐L did not reduce the formation of RORγt^+^ T_regs_ (Figure 2C). In line with IL‐33 being not able to stabilize ICOS‐L expression, we observed a dominant destabilization of ICOS‐L on IL‐3‐stimulated mast cells within 24 h of IL‐33 stimulation (Figure 2D,E). Upon IL‐33 costimulation, the IL‐3‐mediated upregulation of Icosl transcripts was blocked after 24 h and remained at levels comparable to the absence of IL‐3 (Figure 2F). Collectively, we could demonstrate an increase in RORγt^+^ T_reg_ generation by IL‐33‐activated mast cells in the presence of additional IL‐3, which was unexpectedly independent from the IL‐3‐maintained ICOS‐L expression.

### IL‐3 potentiates the IL‐33‐induced IL‐6 and GM‐CSF production

**Figure 3:**
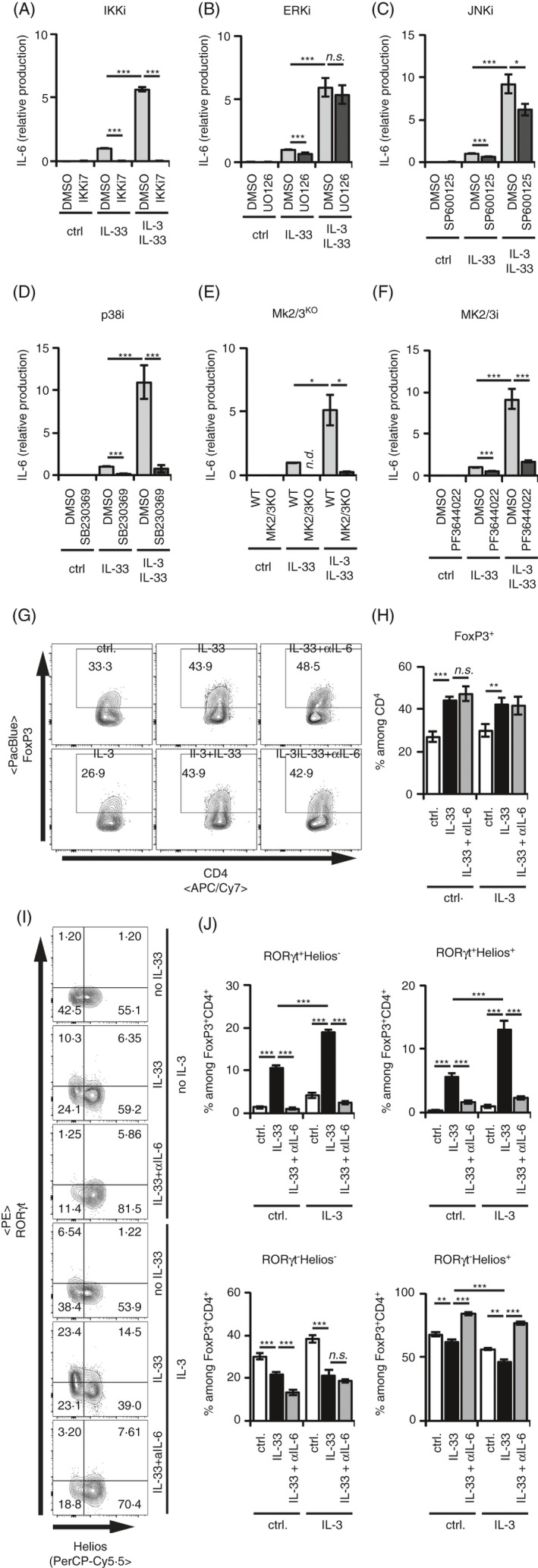
IL‐3‐enhanced IL‐33‐induced RORγt^+^ T_reg_ generation at the expense of RORγt^−^ Helios^+^ T_regs_ is dependent on IKK2 and on the p38‐MK2/3 module. (A‐F) BMMCs were cultured in medium alone or with 50 ng/ml recombinant IL‐33 for 24 h. IL‐6 levels in the supernatants were analysed by ELISA. If indicated, BMMCs were costimulated with 50 ng/ml recombinant IL‐3. All data points were normalized to IL‐33‐induced levels of IL‐6. Data are pooled from independent BMMC cultures. (A) Preincubation of the BMMC with IKKi7 (*n* = 4). (B) Preincubation of the BMMC with UO126 (*n* = 17). (C) Preincubation of the BMMC with SP600125 (*n* = 15). (D) Preincubation of the BMMC with SB203580 (*n* = 10). (E) MK2/3^dKO^ BMMCs were used (*n* = 4). (F) Preincubation of the BMMC with PF3644022 (*n* = 18). (G‐J) CD25^+^ CD4^+^ splenic T_regs_ were cocultured with BMMCs in medium ± 50 ng/ml recombinant IL‐33 alone for 3 days. If indicated, 50 ng/ml recombinant IL‐3 or blocking anti‐IL‐6 antibody was added. Transcription factor expression of FoxP3 among CD4^+^, and RORγt and Helios among FoxP3^+^ CD4^+^ T_regs_ was analysed by flow cytometry in 2 independent experiments with BMMCs from 8 independent donors. (G) Representative FACS plots show FoxP3^+^ cells among CD4^+^ Th cells. (H) Diagram summarizes FoxP3^+^ cells among CD4^+^ Th cells. (I) Representative FACS plots of RORγt^+^ and Helios^+^ cells among FoxP3^+^ CD4^+^ T_regs_. (J) Diagrams summarize RORγt^+^ Helios^−^, RORγt^+^ Helios^+^, RORγt^−^ Helios^+^ and RORγt^−^Helios^−^ cells among FoxP3^+^ CD4^+^ T_regs_. Diagrams show average values of all experiments ± SEM. Statistics were done with Student's *t‐*test: *^n.s^*. not significant; * *p* < 0.05; **p < 0.01, ***p < 0.001

Given that IL‐3 enhanced the IL‐33‐induced and mast cell‐dependent formation of RORγt^+^ T_regs_, we speculated that IL‐3 modulates the IL‐33‐induced cytokine response in mast cells. Confirming and extending our recently published data, IL‐3 potentiated the IL‐33‐induced IL‐6 [Figure 3 and^12^]. This was mediated by IKK2, because inhibition of the kinase with the inhibitor IKK‐16 (IKKiVII^38^) resulted in a complete block of the enhanced IL‐6 response induced by IL‐33 in the presence of IL‐3 (Figure 3A). Different from its role in ICOS‐L surface stabilization (Figure 1J), inhibition of ERK did not show any effect on the IL‐3‐mediated increase in the IL‐33‐induced IL‐6 production (Figure 3B). While inhibition of JNK only showed a very mild reduction of the IL‐3‐mediated enhancement of IL‐6 production (Figure 3C), inhibition of p38 did abolish any IL‐33‐induced effect (Figure 3D). In line with the essential role of the p38‐MK2/3 signalling module for the IL‐33‐induced IL‐6 production in mast cells,^1^, ^21^, the potentiating effect of IL‐3 on the IL‐33‐induced IL‐6 was completely absent in MK2/3 double‐knockout mast cells (Figure 3E). To exclude that this effect is mediated by a destabilization of p38 in MK2/3 double‐knockout mast cells,^39^ we blocked MK2/3 functions with the inhibitor PF3644022^40^ and could reproduce the abolished IL‐6 production of the mutant mast cells (Figure 3F). This demonstrates that the p38/MK2/3 signalling module is essential for mast cells costimulated with IL‐3 and IL‐33.

### IL‐3/IL‐33‐activated mast cells favour the formation RORγt^+^ T_regs_ in dependency of IL‐6

The finding that IL‐3 potentiated the IL‐33‐induced formation of RORγt^+^ T_regs_ by mast cells (Figure 2A,B) and the IL‐33‐induced IL‐6 production (Figure 3A‐E) prompted us to investigate whether the enhancive effect of IL‐3 was dependent on IL‐6. In line with our previous work,^21^ blockade of IL‐6 did neither alter the overall T_reg_ levels in the presence of IL‐33‐stimulated BMMCs nor in the presence of IL‐3‐IL‐33‐costimulated BMMCs (Figure 3G,H). Comparable to the essential role of IL‐6 for the generation of RORγt^+^ T_regs_ by IL‐33‐stimulated mast cells, even that enhanced generation of RORγt^+^ T_regs_ was completely blocked by removal of IL‐6 from the culture (Figure 3I,J). Interestingly, the increase in IL‐33‐induced RORγt^+^ Tregs in the presence of IL‐3 happened at the expense of RORγt^−^ Helios^+^ T_regs_ (Figure 3I,J). Collectively, IL‐6 is also essential for an IL‐3‐mediated increase in RORγt^+^ T_regs_ by IL‐33‐stimulated mast cells.

### IL‐3 blocks the IL‐33‐induced IL‐2 production and reduces the frequency of RORγt^−^Helios^+^ T_regs_

**Figure 4:**
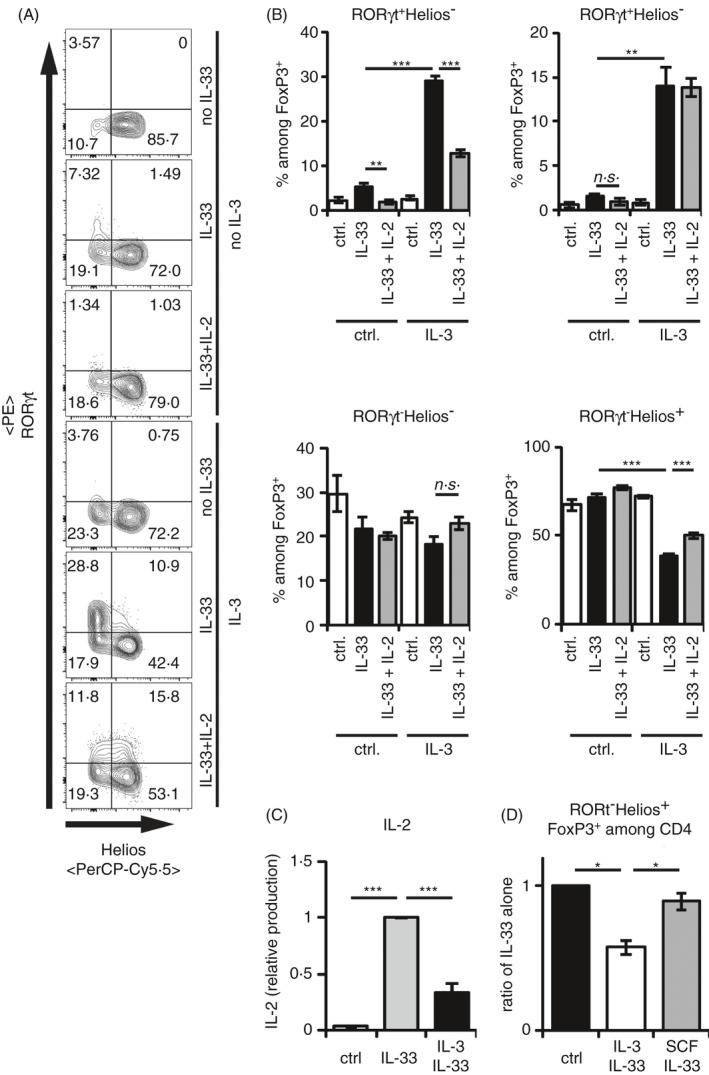
IL‐3 blocks the IL‐33‐induced IL‐2 production by BMMC, which selectively enhances the RORγt^−^ Helios^+^ T_reg_ maintenance. (A, B) CD25^+^CD4^+^ splenic T_regs_ were cocultured with BMMCs in medium ± 50 ng/ml recombinant IL‐33 alone for 3 days. If indicated, 50 ng/ml recombinant IL‐3 or 50 ng/ml recombinant IL‐2 was added. Transcription factor expressions of FoxP3 among CD4^+^, and RORγt and Helios among FoxP3^+^CD4^+^Tregs were analysed by flow cytometry in 2 experiments with BMMCs from 6 independent donors. (A) Representative FACS plots of RORγt^+^ and Helios^+^ cells among FoxP3^+^ CD4^+^ T_regs_. (B) Diagrams summarize RORγt^+^Helios^−^, RORγt^+^Helios^+^, RORγt^−^Helios^+^ and RORγt^−^Helios^−^ cells among FoxP3^+^CD4^+^ T_regs_. (C) BMMCs were cultured in medium alone or with 50 ng/ml recombinant IL‐33 for 24 h. IL‐2 levels in the supernatants were analysed by ELISA. If indicated, BMMCs were costimulated with 50 ng/ml recombinant IL‐3. All data points were normalized to IL‐33‐induced levels of IL‐2. Data were summarized from 4 independent experiments with BMMC of 19 independent donors. (D) Splenic CD25^+^CD4^+^ T_regs_ were cocultured with BMMCs in medium ± 50 ng/ml recombinant IL‐33 alone for 3 days. If indicated, 50 ng/ml recombinant IL‐3 or 50 ng/ml recombinant SCF was added. Transcription factor expressions of RORγt and Helios among FoxP3^+^CD4^+^Tregs were analysed by flow cytometry in 2 experiments with BMMCs from 3 independent donors. Ratio of RORγt^−^Helios^+^ Tregs among Th cells related to conditions with IL‐33 alone is shown. Diagrams show average values of all experiments ± SEM. Statistics were done with Student's *t‐*test: *^n.s^*. not significant; * *p* < 0.05; **p < 0.01, ***p < 0.001

A maintenance factor for Helios^+^ T_regs_ is IL‐2.^21^ To investigate whether the reduction of RORγt^−^ Helios^+^ T_regs_ can be rescued by the addition of IL‐2, we added IL‐2 to the coculture of IL‐3‐IL‐33‐stimulated BMMCs and T_regs_. Interestingly, the addition of IL‐2 recovered the maintenance of RORγt^−^ Helios^+^ T_regs_ in the presence of IL‐3 and IL‐33 (Figure 4A,B). This suggested that the reduction in RORγt^−^Helios^+^ T_regs_ resulted from a deficiency of IL‐2 in the culture, which is normally produced by IL‐33‐stimulated BMMCs.^21^ In contrast to conditions with SCF costimulation,^21^ IL‐3 strongly reduced the IL‐33‐induced production of IL‐2 by mast cells (Figure 4C). In line with its lacking suppressive effect on the IL‐33‐induced IL‐2 secretion by mast cells,^21^ SCF costimulation did not reduce the RORγt^−^Helios^+^ T_regs_ in the presence of IL‐33‐stimulated BMMCs (Figure 4D). Collectively, these data demonstrate that different from SCF, the presence of IL‐3 alters the cytokine profile induced by IL‐33‐activated mast cells and therefore enhanced the shift of the balanced formation of RORγt^+^ T_regs_ and Helios^+^ T_regs_ towards RORγt^+^ T_regs_.

## DISCUSSION

Here, we demonstrate that IL‐3 strongly impacts the IL‐33‐induced cytokine profile in mast cells. Costimulation with IL‐3 and IL‐33 promotes p38‐MK2/3‐mediated effector functions. This results in a potentiated IL‐6, but an inhibited IL‐2 response.

The IL‐33‐induced IL‐6 production is mediated by the TAK1‐IKK2‐IκB‐p65 signalling pathway.^22^ While IL‐3 alone does not activate NFκB per sé,^12^ the enhanced production of IL‐6 by costimulation with IL‐3 and IL‐33 suggests that IL‐3 modulated the NFκB activity. In earlier studies, we could show that SCF enhances the IL‐33‐induced activation of the TAK/IKK complex, which results in an increase in the activities of NFκB, and ultimately enhanced the production of IL‐6.^22^, ^23^ Therefore, we suggest that IL‐3 also enhances the IL‐33‐induced activation of TAK1/IKK2 complex, which leads to an increased destabilization of IκB^12^ and therefore primes NFκB for enhanced activation. While the IL‐33‐induced production of IL‐6 in the presence of IL‐3 was potentiated independently from ERK1/2 and JNK1/2, it was mediated via the p38‐MK2/3 signalling module similar to stimulation with IL‐33 alone.^21^ It is widely accepted that IL‐3 activates p38 in mast cells,^41^ but it is not entirely clear whether p38 is activated by TAK‐1 via MKKs^42^ or whether it is subjected to interaction with a TAK‐IKK‐TPL2 complex.^43^

In contrast to the enhanced IL‐6 production, IL‐3‐mediated mechanism of costimulation did not result in a potentiated IL‐33‐induced IL‐2 production. Notably, IL‐3 even inhibited the IL‐33‐induced IL‐2 production. The mechanism how IL‐3 counter‐regulated the IL‐33‐induced ERK1/2‐dependent IL‐2 production remains unknown. An autocrine feedback loop via IL‐2‐IL2R activation is highly unlikely, because mast cells express the IL‐2Rα and the IL‐2Rγ chain, but not the IL‐2Rβ chain, which is essential for IL‐2 signalling.^44^ However, IL‐33 induces expression of the IL‐33‐induced ERK1/2‐specific dual‐specificity phosphatase 5 (DUSP5) in eosinophils.^45^ DUSP5 has been demonstrated to negatively regulate the ERK activity,^46^ which is essential for the IL‐33‐mediated induction of IL‐2 in mast cells.^21^ Therefore, as a potential mechanism we suggest an enhanced expression of DUSP5 in IL‐3/IL‐33‐stimulated BMMCs, which in turn controls the production of IL‐2 via deactivation of ERK1/2.

We recently showed that among mast cell produced cytokines, SCF potentiates both IL‐33‐induced IL‐6 and IL‐2.^21^ According to this, SCF increased the formation of RORγt^+^ T_regs_, whereas the maintenance of Helios^+^ T_regs_ is not affected.^21^ In the absence of IL‐3, IL‐33‐activated mast cells mediate a balanced production of IL‐6 and IL‐2 and thus regulate the dichotomy between RORγt^+^ T_regs_ and Helios^+^ T_regs_.^21^ Therefore, mast cell‐derived IL‐6 promotes the formation and stability of RORγt^+^ T_regs_, whereas the produced IL‐2 maintains HELIOS^+^ T_regs_.^21^ In the presence of IL‐3, the IL‐33‐induced IL‐6 response is potentiated and the production of IL‐2 is blocked. This altered cytokine profile correlated with an enhanced shift of the dichotomy between RORγt^+^ T_regs_ and HELIOS^+^ T_regs_ towards RORγt^+^ T_regs_ (Figure S3).

Recently, Kim et al. showed that ICOS‐L on DCs is essential to maintain the formation of RORγt^+^ T_regs_.^36^ The expression of ICOS‐L on mast cells is dependent on IL‐3 and is mediated by MAPK signalling via ERK, but not by p38 or JNK (Figure S3A). However, when we tested the role of ICOS‐L by using a blocking ICOS‐L antibody, we did not detect any influence on the formation of RORγt^+^ T_regs_ or maintenance of HELIOS^+^ T_regs_. Concomitantly, we detected a strong downregulation of ICOS‐L on BMMCs in the presence of IL‐33, thereby explaining the absence of a blocking effect of ICOS‐L. This indicated that ICOS‐L is not involved in the IL‐33‐induced mast cell‐mediated dichotomy between RORγt^+^ and Helios^+^ T_regs_. Surprisingly, we did not observe any strong change in FoxP3 expression within the T_reg_ compartment depending on the presence of IL‐3. Although IL‐3 is considered as a survival factor for mast cells,^47^ the cell viability was hardly reduced during most of the coculture period, and therefore, increased mast cell viability would not account for the effect of IL‐3 on the RORγt^+^ Treg. These observations suggest that IL‐33 stimulation maintains T_regs_ mainly via factors different from ICOS‐L or IL‐2. Considering the alarmin function of IL‐33, one could speculate that ICOS‐L‐ or IL‐2‐driven regulatory mechanisms are blocked in situations of tissue destruction, but by generation of RORγt^+^ T_regs_, the shutdown of the damage‐induced immune response would be initiated.

Being effective in protection from colitis, RORγt^+^ T_regs_ have been established to reflect a key T_reg_ subpopulation.^48^, ^49^ However, the formation of RORγt^+^ T_regs_ *in vivo* has been shown to essentially depend on IL‐6, for example in the gastrointestinal tract.^50^, ^51^ As a modulator of intestinal inflammatory diseases, IL‐33 is released during inflammation and controls the severity of the immune response via the activation of mast cells.^52^, ^53^ In such a context, SCF might be secreted, which not only facilitates the differentiation and survival of mast cells, but also might control their responses during the recovery phase upon inflammation.^53^ In the presented work, we addressed another important factor in mast cell biology different from SCF: IL‐3. By a yet unknown selective impact on the cytokine production, IL‐3 did enhance IL‐6, but decreased IL‐2 production induced by IL‐33 (Figure S3B,C). That appeared to be different from SCF costimulation that not only enhanced IL‐33‐induced formation of RORγt^+^ T_regs_, but also did not reduce IL‐2 production by mast cells^21^ and therefore maintained Helios^+^ T_regs_. Taken together, these observations indicate that IL‐3 facilitates the IL‐33‐dependent mast cell activation different from SCF resulting in altered mast cell‐dependent modulation of RORγt^+^ T_regs_ or HELIOS^+^ T_reg_ responses to tissue injury (Figure S3C). IL‐3 is mainly produced by T cells,^13^ which preferentially locate in skin or mucosal tissues and can be activated by the alarmin IL‐33.^54^ Therefore, tissue‐prevalent mast cells stimulated by a combination of IL‐3 and IL‐33 would secrete high levels of IL‐6, but not IL‐2, and represent potential mediators of the negative regulatory role of IL‐3 in colitis‐mediated immune responses by induction of RORγt^+^ Tregs.^48^ Given the specific modulatory effects of IL‐3 on mast cell function, the manipulation of IL‐3 levels in mast cell‐mediated responses should be considered for treatment of inflammatory diseases.

## FUNDING INFORMATION

This work was supported by the grant from Deutsche Forschungsgemeinschaft (DFG DR 1113/1‐1 to S.D.) and by the Interdisziplinäres Zentrum für klinische Forschung, Jena (IZKF—MSP1 Medical Scientist Program to N.A.).

## CONFLICT OF INTEREST

The authors declare no competing financial interests. The authors did not have any discussions with an *Immunology* Editor about the work described in this manuscript. The work has not been published elsewhere.

## AUTHOR CONTRIBUTIONS

S.D. developed the concept, designed the research, performed experiments, analysed data, generated figures and wrote the manuscript; S.M., F.W., P.W. and R.B‐L. performed experiments and analysed data; M.G. provided the mk2^−/−^/mk3^−/−^ double‐deficient mouse line; A.H. and T.K. provided essential reagents; and N.A. developed the concept, designed the research, performed experiments, analysed data, generated figures and wrote the manuscript.

## Supporting information

Figure S1. Gating strategy for the flow cytometrical analysis of BMMCs and T_regs_. Cellular events were defined in the FSC‐A/SSC‐A plot. Subsequently, among these gated cells, singlets were identified via FSC‐H/FSC‐W and SSC‐H/SSC‐W analysis. (A) Mast cells were identified among living cells (DAPI^−^) via the expression of CD117. (B) FoxP3^+^CD4^+^ Tregs were identified among CD4^high^SSC‐A^low^ culture cells via the expression of FoxP3.

Figure S2. BMMC survival starts to decrease after 48 h of IL‐3 deprivation. BMMCs were washed and cultured in medium alone or with 50 ng/ml recombinant IL‐33 for 72 h. If indicated, 50 ng/ml recombinant IL‐3 was added to the cultures. After 24 h, 48 h and 72 h the frequencies of DAPI^+^ cells among the CD117^+^ cells were analysed by flowcytometry. FACS plot show representative results for the indicated conditions (A). Data from 3 independent biological replicates are shown in (B).

Figure S3. Model of the interaction of IL‐3 and IL‐33 as presented in the manuscript. (A) IL‐3 is essential for the surface expression of ICOS‐L via ERK1/2. (B) IL‐33 induces the production of IL‐6 and IL‐2 via TAK1‐IKK2 signalling and via TAK1‐p38‐MK2/3 signalling. While IL‐6 is important for the induction of RORγt^+^ Tregs, IL‐2 supports the stability of Helios^+^ Tregs. (C) We presented that IL‐33 inhibits the IL‐3‐induced ICOS‐L expression. Via a yet unknown mechanism, IL‐3 did almost completely block the IL‐2 production induced by IL‐33 but potentiated the production of IL‐6. Consequently, IL‐3 shifted the IL‐33‐induced T_reg_dichotomy towards RORγt^+^ T_regs_at the expense of RORγt^‐^ Helios^+^ T_regs_.

## Data Availability

Data relating to the manuscript will be available upon reasonable request to the corresponding authors.
