# Supplementary figures and images for: IL‐3 is essential for ICOS‐L stabilization on mast cells, and sustains the IL‐33‐induced RORγt+ Treg generation via enhanced IL‐6 induction
Source: Immunology. 2021 Jan 27;163(1):86–97. doi: 10.1111/imm.13305 (PMC8044339; doi:10.1111/imm.13305)

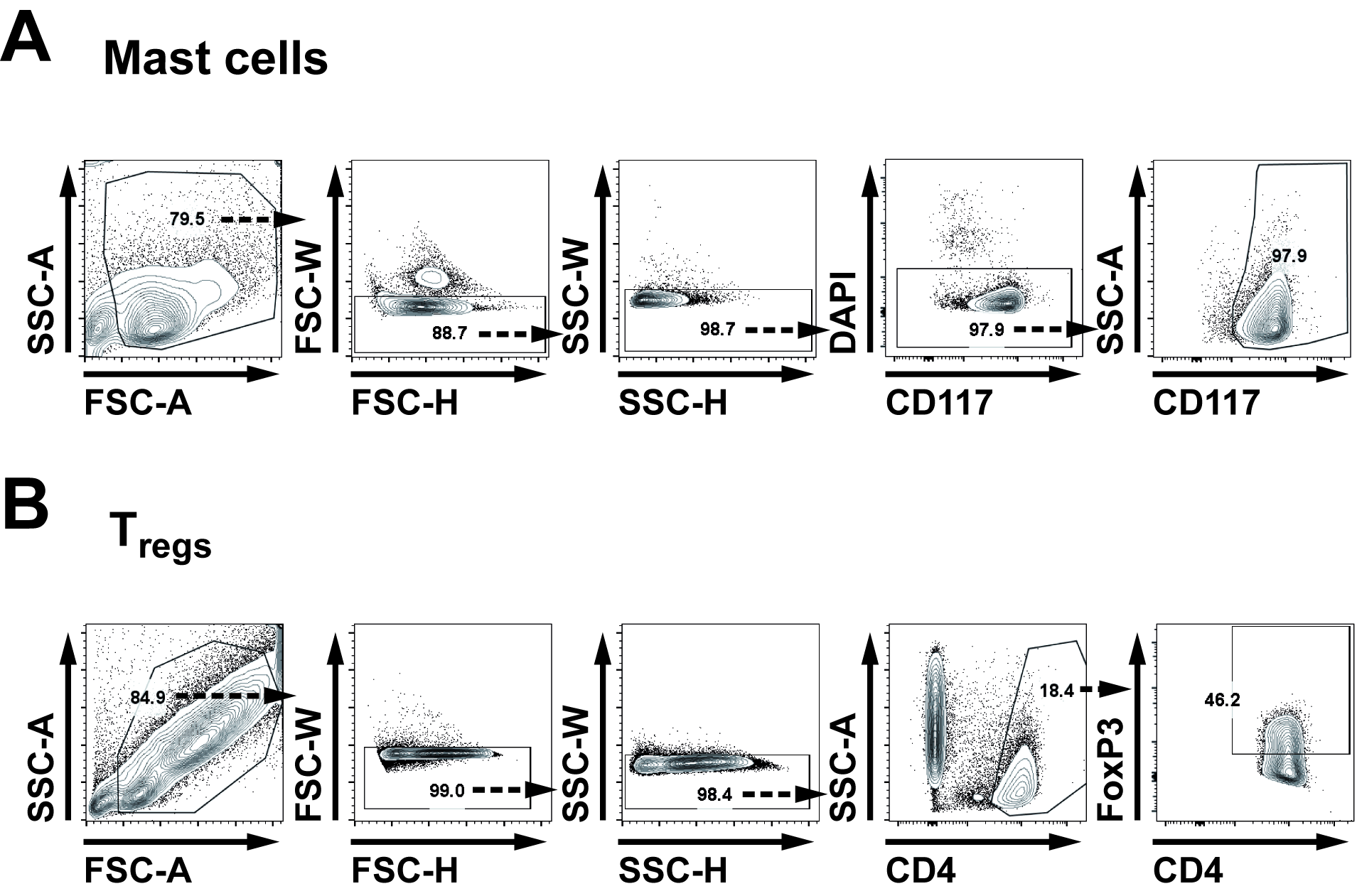

Supplement: Supplementary file 1 — Figure S1. Gating strategy for the flow cytometrical analysis of BMMCs and Tregs. Cellular events were defined in the FSC‐A/SSC‐A plot. Subsequently, among these gated cells, singlets were identified via FSC‐H/FSC‐W and SSC‐H/SSC‐W analysis. (A) Mast cells were identified among living cells (DAPI−) via the expression of CD117. (B) FoxP3+CD4+ Tregs were identified among CD4highSSC‐Alow culture cells via the expression of FoxP3. [file IMM-163-86-s003.tif]

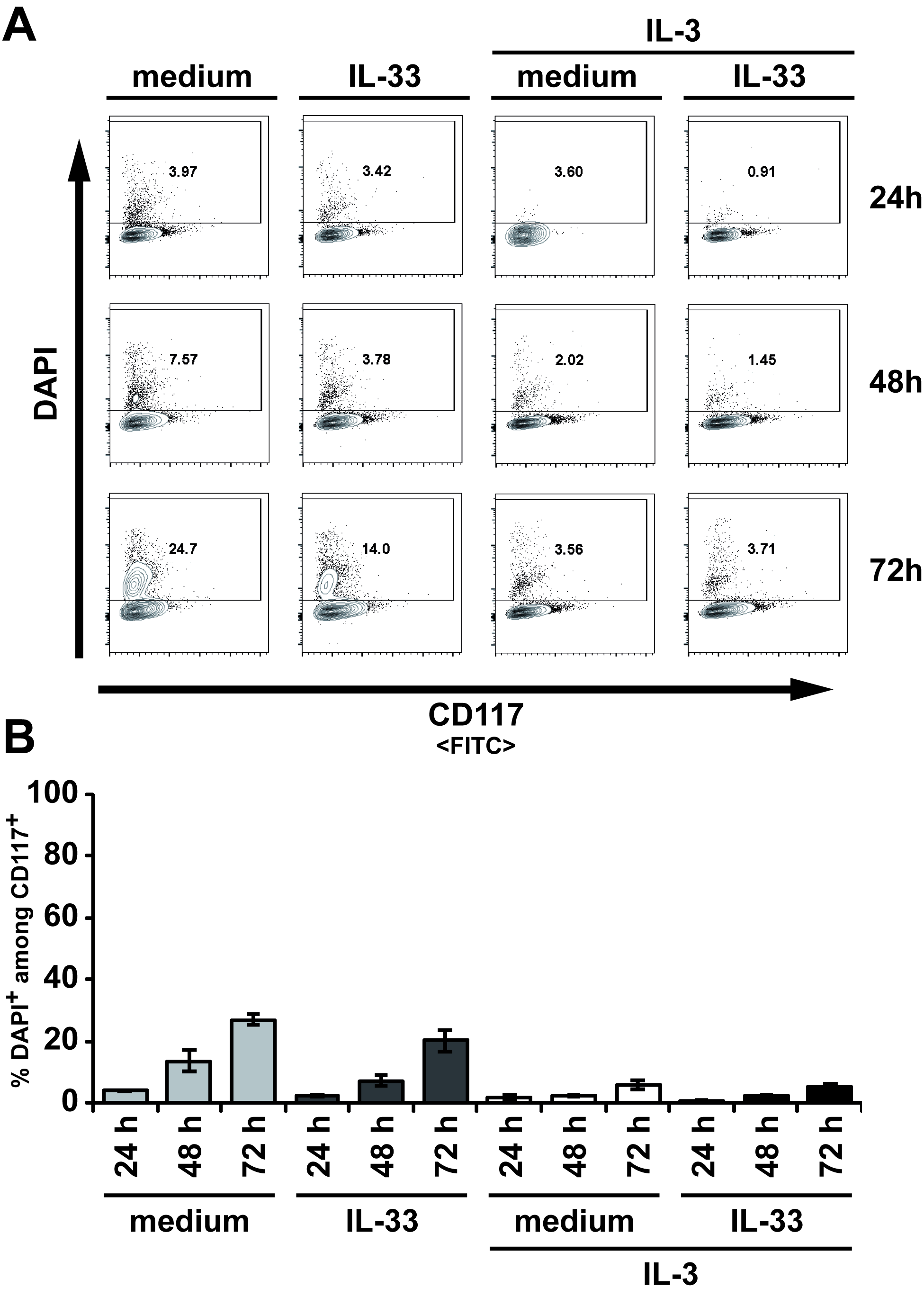

Supplement: Supplementary file 2 — Figure S2. BMMC survival starts to decrease after 48 h of IL‐3 deprivation. BMMCs were washed and cultured in medium alone or with 50 ng/ml recombinant IL‐33 for 72 h. If indicated, 50 ng/ml recombinant IL‐3 was added to the cultures. After 24 h, 48 h and 72 h the frequencies of DAPI+ cells among the CD117+ cells were analysed by flowcytometry. FACS plot show representative results for the indicated conditions (A). Data from 3 independent biological replicates are shown in (B). [file IMM-163-86-s001.tif]

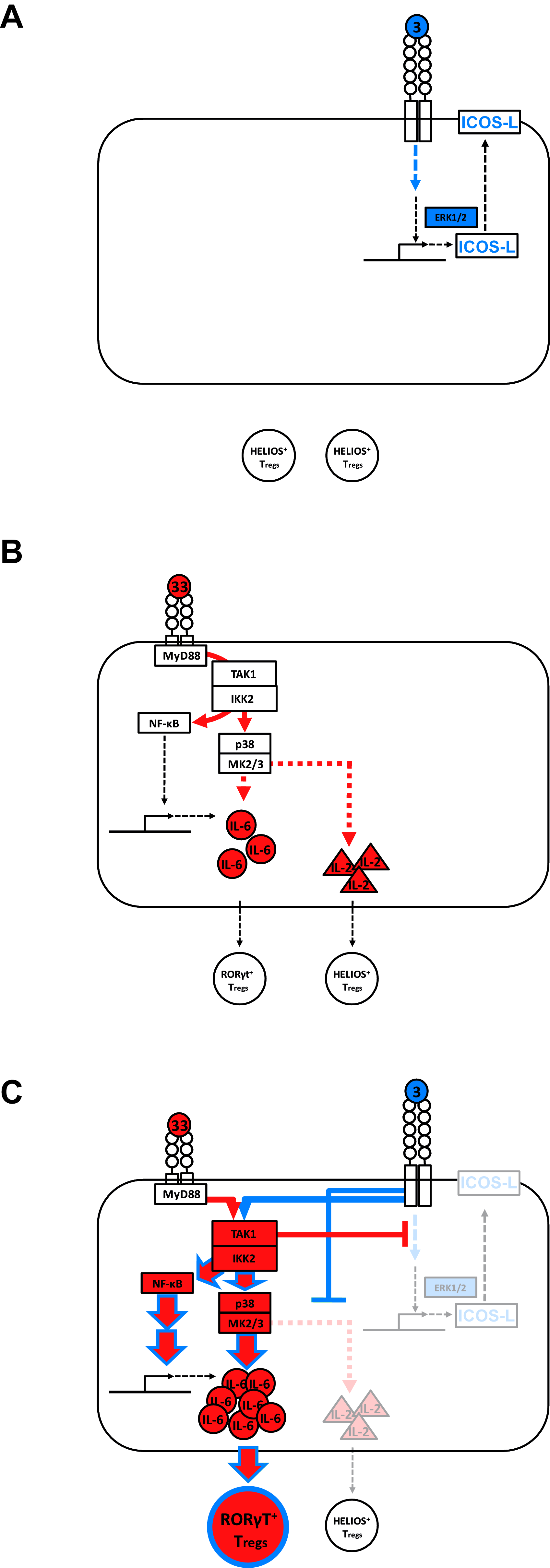

Supplement: Supplementary file 3 — Figure S3. Model of the interaction of IL‐3 and IL‐33 as presented in the manuscript. (A) IL‐3 is essential for the surface expression of ICOS‐L via ERK1/2. (B) IL‐33 induces the production of IL‐6 and IL‐2 via TAK1‐IKK2 signalling and via TAK1‐p38‐MK2/3 signalling. While IL‐6 is important for the induction of RORγt+ Tregs, IL‐2 supports the stability of Helios+ Tregs. (C) We presented that IL‐33 inhibits the IL‐3‐induced ICOS‐L expression. Via a yet unknown mechanism, IL‐3 did almost completely block the IL‐2 production induced by IL‐33 but potentiated the production of IL‐6. Consequently, IL‐3 shifted the IL‐33‐induced Tregdichotomy towards RORγt+ Tregsat the expense of RORγt‐ Helios+ Tregs. [file IMM-163-86-s002.tif]
